# Mitochondrial reactive oxygen species-mediated NLRP3 inflammasome activation contributes to aldosterone-induced renal tubular cells injury

**DOI:** 10.18632/oncotarget.8243

**Published:** 2016-03-21

**Authors:** Wei Ding, Honglei Guo, Chengyan Xu, Bin Wang, Minmin Zhang, Feng Ding

**Affiliations:** ^1^ Division of Nephrology, Shanghai Ninth People's Hospital, School of Medicine, Shanghai Jiaotong University, Shanghai, China; ^2^ Division of Nephrology, The Fifth People's Hospital of Shanghai, Fudan University, Shanghai, China; ^3^ Division of Nephrology, Huashan Hospital and Institute of Nephrology, Fudan University, Shanghai, China

**Keywords:** aldosterone, mitochondrial reactive oxygen species, NLRP3 inflammasome, renal tubular cells, chronic kidney disease, Pathology Section

## Abstract

Aldosterone (Aldo) is an independent risk factor for chronic kidney disease (CKD), and although Aldo directly induces renal tubular cell injury, the underlying mechanisms remain unclear. NLRP3 inflammasome and mitochondrial reactive oxygen species (ROS) have recently been implicated in various kinds of CKD. The present study hypothesized that mitochondrial ROS and NLRP3 inflammasome mediated Aldo–induced tubular cell injury. The NLRP3 inflammasome is induced by Aldo in a dose- and time-dependent manner, as evidenced by increased NLRP3, ASC, caspase-1, and downstream cytokines, such as interleukin (IL)-1β and IL-18. The activation of the NLRP3 inflammasome was significantly prevented by the selective mineralocorticoid receptor (MR) antagonist eplerenone (EPL) (*P* < 0.01). Mice harboring genetic knock-out of NLRP3 (NLRP3^−/−^) showed decreased maturation of renal IL-1β and IL-18, reduced renal tubular apoptosis, and improved renal epithelial cell phenotypic alternation, and attenuated renal function in response to Aldo-infusion. In addition, mitochondrial ROS was also increased in Aldo-stimulated HK-2 cells, as assessed by MitoSOX^TM^ red reagent. Mito-Tempo, the mitochondria-targeted antioxidant, significantly decreased HK-2 cell apoptosis, oxidative stress, and the activation of NLRP3 inflammasome. We conclude that Aldo induces renal tubular cell injury *via* MR dependent, mitochondrial ROS-mediated NLRP3 inflammasome activation.

## INTRODUCTION

Aldosterone (Aldo) is produced in the adrenal zona glomerulosa, and mediates salt and water homeostasis by interaction with mineralocorticoid receptors (MR), which are expressed in renal epithelial cells [1-3]. Recently, a growing body of evidence indicates that the direct action of Aldo on the MR plays a key role in the progression of chronic kidney disease (CKD). Exogenous infusion of Aldo was able to reverse the protective effects of angiotensin II blockade in the remnant kidney rat model [4, 5]. Moreover, rats receiving MR antagonist displayed significantly attenuated renal injury induced by cyclosporine A and radiation [6, 7]. Recently, tubular cell injury has been suggested to be an important mechanism for the progression of CKD, which ultimately leads to tubulointerstitial fibrosis. Tubular cell apoptosis was demonstrated to be involved in the progression of polycystic kidney disease [8]. Yuan *et al.* demonstrated that Aldo induced epithelial-to-mesenchymal transition (EMT) and renal proximal tubular cells injury *via* mitochondrial dysfunction [9]. Patni *et al.* also showed that Aldo induced tubular epithelial cell apoptosis through the excessive production of reactive oxygen species (ROS) [2]. These data suggested a contribution of Aldo to the progression of renal tubular injury. However, the underlying mechanism of Aldo-induced renal tubular injury is still not fully understood.

Accumulating evidence suggests that inflammation in the absence of pathogens, referred to as sterile inflammation, mediated *via* the inflammasome. Nucleotide-binding domain and leucine-rich repeat containing PYD-3 (NLRP3) is the best characterized inflammasome. The NLRP3 inflammasome is a cytoplasmic protein complex that is activated upon signs of cellular ‘danger’ to trigger innate immune defenses. Upon detecting cellular stress, NLRP3 recruits the adaptor protein ASC and procaspase-1, which results in caspase-1 activation and processing of cytoplasmic targets, including interleukin (IL)-1β and IL-18 [10, 11]. Recently, the NLRP3 inflammasome was demonstrated to participate in the pathogenesis of kidney disease. NLRP3 deficiency was shown to ameliorate renal inflammation and fibrosis in a model of renal tubular injury [12, 13]. These studies suggest that the NLRP3 inflammasome is involved in the development of various kinds of CKD.

Several mechanisms underlying the activation of the NLRP3 inflammasome have been demonstrated, including ion channel gating, lysosome rupture, and excessive ROS generation. The notion of activation of the NLRP3 inflammasome by increased ROS production was widely accepted and it was suggested that the NLRP3 inflammasome may be a general sensor for changes in cellular oxidative stress [14, 15]. Additionally, the role of mitochondria as the main source of cellular ROS has also been explored. Various triggers induce mitochondrial ROS generation, such as hypoxia, cell membrane damage, and increased metabolic rates [16]. Zhang *et al.* showed that Aldo induced EMT *via* mitochondrial ROS in renal tubular epithelial cells [3]. However, the role of the NLRP3 inflammasome activation and its correlation with mitochondrial ROS in Aldo-induced renal tubular injury still remains unknown. The purpose of this study was to investigate whether mitochondrial ROS-mediated the activation of NLRP3 inflammasome that contributes to Aldo-induced renal tubular injury.

## RESULTS

### Aldosterone induces NLRP3 inflammasome activation in HK-2 cells

**Figure 1:**
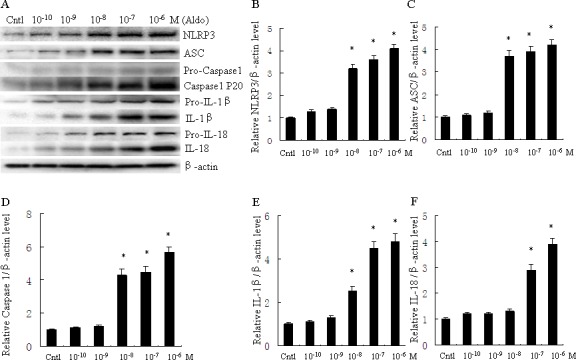
Aldo dose-dependent activated NLRP3 inflammasome in HK-2 cells **A.** Western blot of NLRP3, ASC, activated caspase-1, IL-1β, and IL-18. HK-2 cells were treated with Aldo (ranging from 10^−10^-10^−6^ M) for 24 h. **B.-F.** densitometric analysis of protein expression of NLRP3 (B), ASC (C), active caspase-1 (D), IL-1β (E), and IL-18 (F). Data are expressed as mean ± SEM (*n* = 6). *, *P* < 0.01 *vs*. Cntl. Cntl: untreated control group.

**Figure 2:**
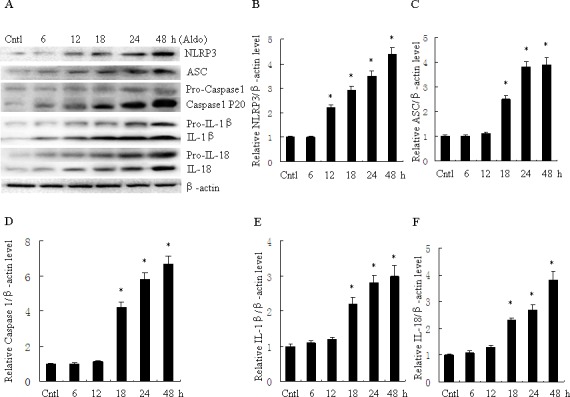
Aldo time-dependent activated NLRP3 inflammasome in HK-2 cells **A.** Western blot of NLRP3, ASC, activate caspase-1, IL-1β, and IL-18. HK-2 cells were treated with Aldo (10^−7^ M) for 6-48 h. **B.**-**F.** densitometric analysis of NLRP3 (B), ASC (C), active caspase-1 (D), IL-1β (E), and IL-18 (F). Data are expressed as mean ± SEM (*n* = 6). *, *P* < 0.01 *vs*. Cntl. Cntl:untreated control group.

To evaluate activation of the NLRP3 inflammasome in Aldo-treated tubular epithelial cells, we assessed the markers of inflammasome activation including NLRP3, ASC, posttranslational processing of caspase-1 (cleaved caspase-1, Caspase-1 P20), and the proinflammatory cytokines IL-1β and IL-18. As shown in Figure 1, protein expression of NLRP3, ASC, caspase-1, IL-1β and IL-18 significantly increased following Aldo stimulation of HK-2 cells for 24 h, in a dose-dependent manner. Similarly, protein expression of NLRP3, ASC, caspase-1, IL-1β and IL-18 were markedly increased in HK-2 cells after treatment with Aldo in a time-dependent manner (Figure 2). The increased expression of these proteins was detected as early as 12 h after treatment with Aldo (10^−7^ M). These results indicate that Aldo treatment triggered activation of the NLRP3 inflammasome in HK-2 cells.

### SiNLRP3 attenuates Aldo-induced activation of the NLRP3 inflammasome in HK-2 cells

**Figure 3:**
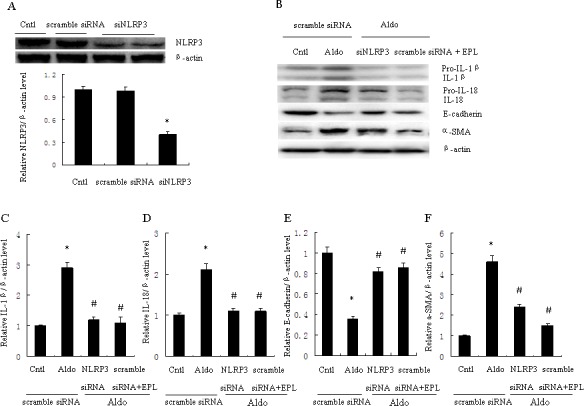
Transfection with siNLRP3 or treatment with EPL inhibits Aldo-induced NLRP3 inflammasome and phenotypic alternation in HK-2 cells **A.** Western blot of NLRP3 protein expression. Cells were transfected with scramble siRNA or siNLRP3 (siRNA against NLRP3). **B.** Western blot of IL-1β, IL-18, E-cadherin, and α-SMA. HK-2 cells were transfected with 500 nM siNLRP3 or scramble siRNA for 24 h before treatment with Aldo (10^−7^ M). At the end of the incubation period, NLRP3 inflammasome activation and cell phenotypic alternation was evaluated. **C.**-**F.** densitometric analysis of IL-1β (C), IL-18 (D), E-cadherin (E) and α-SMA (F). Data are expressed as mean ± SEM (*n* = 6). *, *P* < 0.01 *vs*. Cntl+ scramble siRNA; ^#^, *P* < 0.01 *vs*. Aldo+scramble siRNA group. Cntl: untreated control group.

NLRP3 inflammasome activation induced posttranslational processing of caspase-1 and the proinflammatory cytokines IL-1β and IL-18. HK-2 cells were transfected with scramble siRNA or siRNA against NLRP3 (siNLRP3), and protein expression of mature IL-1β and IL-18 was evaluated. Treatment with 500 nM siNLRP3 resulted in a 60% reduction of NLRP3 protein expression in HK-2 cells (Figure 3A). Knockdown of NLRP3 also significantly decreased Aldo-dependent induction of mature IL-1β and IL-18 protein expression (Figure 3B). Similarly, the MR antagonist EPL also markedly reduced Aldo-dependent induction of IL-1β and IL-18 protein expression (Figure 3B-3D).

### SiNLRP3 attenuates Aldo-induced phenotypic alternation and apoptosis

**Figure 4:**
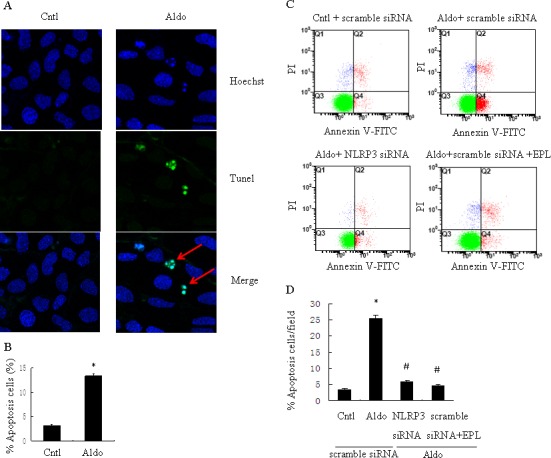
siNLRP3 and EPL inhibit Aldo-induced apoptosis in HK-2 cells **A.** Representative images of Hoechst 33258 and TUNEL staining in control HK-2 cells or HK-2 cells treated with Aldo. Arrows indicate apoptotic cells. **B.** Quantitation of apoptosis. The cells were stained and determined by flow cytometry. **C.** HK-2 cells were transfected with 500 nM siNLRP3 or scramble siRNA for 24 h before treatment with Aldo (10^−7^ M). **D.** HK-2 cell apoptosis was measured by flow cytometry. At the end of the incubation period, apoptosis was evaluated in the cells. Data are expressed as mean ± SEM (*n* = 6). *, *P* < 0.01 *vs*. Cntl; ^#^,*P* < 0.01 *vs*. Aldo+scramble siRNA group. Cntl: untreated control group.

To further examine the role of NLRP3 in the phenotypic alternation of HK-2 cells induced by Aldo-treatment, we analyzed E-cadherin and α-SMA protein expression by western blot. E-cadherin protein expression was significantly decreased and a-SMA protein expression was significantly increased in Aldo-treated group compared to the control HK-2 cells (Figure 3B, 3E, and 3F). In contrast, treatment with siNLRP3 or EPL markedly attenuated loss of E-cadherin and induction of α-SMA protein expression (Figure 3B, 3E and 3F). We also examined the role of NLRP3 in the induction of apoptosis of HK-2 cells. Apoptosis was detected using flow cytometric methods to assess Annexin-V, propidium iodide, and TUNEL assays. The apoptosis of HK-2 cells in the Aldo-treated group was significantly higher than the control group (Figure 4A, 4B), and the induction of apoptosis was markedly inhibited by treatment with siNLRP3. Moreover, EPL also inhibited HK-2 cell apoptosis (Figure 4C, 4D). These results indicate that Aldo induces renal tubular cell injury *via* an MR-dependent pathway which is mediated through activation of the NLRP3 inflammasome.

### Deletion of NLRP3 inflammasome in mice ameliorates Aldo-induced renal injury

**Figure 5:**
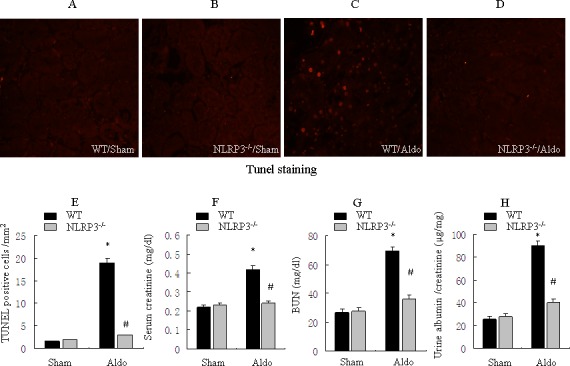
Knockout of NLRP3 ameliorates Aldo-induced tubular epithelial cell apoptosis and renal function in mice treated with Aldo-infusion **A.**-**D.** Kidney cortical tissue was examined by TUNEL staining to evaluate tubular epithelial cells apoptosis, representative images of TUNEL staining at 200X magnification are shown. **E.** Quantification of TUNEL-positive cells in the tissues from each treatment group. F-H: Analysis of physiological parameters, including serum creatinine (F), BUN (G), and urine albumin/creatinine ratio (H). Data are expressed as means ± SEM (*n* = 6). *, *P* < 0.01 *vs*. WT/Sham group; ^#^, *P* < 0.01 *vs*. WT/Aldo group.

We investigated the role of NLRP3 inflammasome in the pathophysiological feature of Aldo-induced renal injury *in vivo*. As shown in Figure 5, Very little apoptosis in tubular cells was detected in the kidneys of WT/sham or NLRP3^−/−^/sham mice (Figure 5A, 5B, 5E). However, after mice were infused with Aldo, significant apoptosis was showed in kidney cortical tissues in the WT/Aldo group (Figure 5C, 5E). Importantly, much less apoptosis was measured in tubular cells of the NLRP3^−/−^/Aldo group (Figure 5D, 5E). Serum creatinine and BUN levels were markedly elevated in WT/Aldo mice compared to WT/sham mice. However, serum creatinine and BUN levels were reduced in NLRP3^−/−^/Aldo mice compared to WT/Aldo mice (Figure 5F and 5G). Similarly, the urine albumin/creatinine ratio was increased in WT/Aldo mice relative to WT/sham mice; the increased urine albumin/creatinine ratio was moderately recovered in the NLRP3^−/−^/Aldo mice (Figure 5H).

### Deletion of NLRP3 inflammasome in mice ameliorates Aldo-induced inflammatory cytokine expression and phenotypic alternation

**Figure 6:**
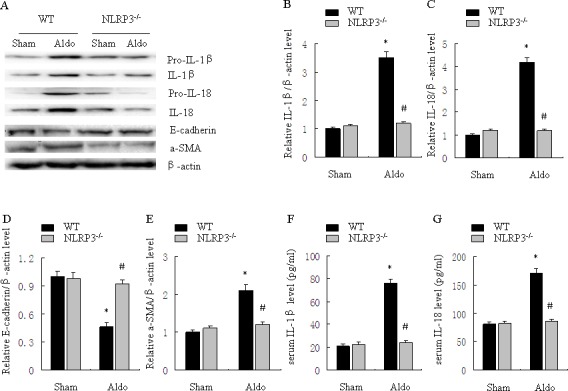
Knockout of NLRP3 ameliorates Aldo-induced activation of the NLRP3 inflammasome and phenotypic alternation in mice treated with Aldo-infusion **A.** Western blot of IL-1β, IL-18, E-cadherin, and α-SMA. **B.**-**E.** Densitometric analysis of IL-1β (B), IL-18 (C), E-cadherin (D), and α-SMA (E) expression. **F.**, **G.** ELISA analysis of serum IL-1β (F) and IL-18 (G) concentration. Data are expressed as mean ± SEM (*n* = 6). *, *P* < 0.01 *vs*. WT/Sham group; ^#^, *P* < 0.01 *vs*. WT/Aldo group.

As shown in Figure 6, renal IL-1β and IL-18 protein were significantly increased in WT/Aldo mice compared with WT/sham mice. Importantly, Aldo-dependent induction of renal IL-1β and IL-18 protein was inhibited in NLRP3^−/−^/Aldo mice (Figure 6A-6C). Serum IL-1β and IL-18 protein secretion were also reduced in NLRP3^−/−^/Aldo mice (Figure 6F, 6G). Compared with WT/sham mice, renal E-cadherin protein was reduced and renal α-SMA was significantly increased in WT/Aldo mice. However, these indicators of phenotypic alternation were not observed in the NLRP3^−/−^/Aldo mice (Figure 6D, 6E).

### Aldo induces mitochondrial ROS production *in vitro*

**Figure 7:**
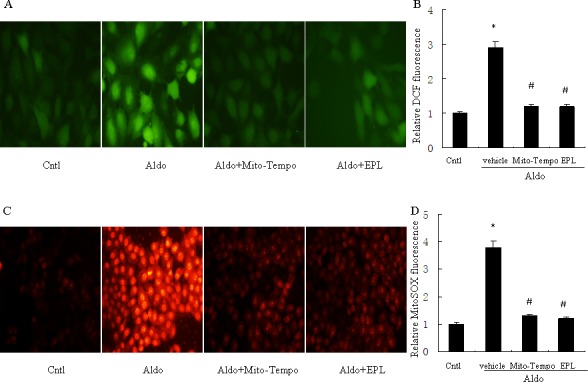
Treatment with Mito-Tempo or EPL prevents Aldo-induced mitochondrial ROS production For detection of ROS, HK-2 cells were seeded into six well plates, and allowed to grow until they reached confluent cell density, they were washed twice with PBS and incubated with DCFDA (10 μM) or MitoSOX (5 μM) for 30 min, then treated with Aldo (10^−7^ M) in the presence of Mito-Tempo (10 μM), EPL (10 μM), or vehicle. **A.** Representative images of HK-2 cells stained with dichlorodihydrofluorescein diacetate. **B.** Quantification of 2′,7′-dichlorofluorescein (DCF) fluorescence. **C.** Representative images of HK-2 cells stained with MitoSOX. **D.** Quantification of MitoSOX fluorescence. Data are expressed as mean ± SEM (*n* = 6). *, *P* < 0.01 *vs*. Cntl ; ^#^, *P* < 0.01 *vs*. Aldo-treated group.

Our previous study indicated the involvement of ROS in Aldo-induced renal injury [17], thus we then tested whether mitochondrial ROS is triggered by Aldo in tubular epithelial cells, ROS production was markedly increased by Aldo treatment in HK-2 cells, as visualized by fluorescence microscopy, while treatment with the mitochondria-targeted antioxidant Mito-Tempo, markedly abolished the Aldo-induced increased ROS levels (Figure 7A, 7B). Additionally, Aldo triggered mitochondrial ROS generation, as detected by MitoSOX^TM^ Red, which was significantly blunted by Mito-Tempo (Figure 7C, 7D). EPL also markedly inhibited ROS production in Aldo-stimulated HK-2 cells (Figure 7B, 7D), suggesting that the Aldo induced mitochondrial ROS generation is mediated *via* MR.

### Inhibition of mitochondrial ROS attenuates apoptosis and phenotypic alternation in HK-2 cell

**Figure 8:**
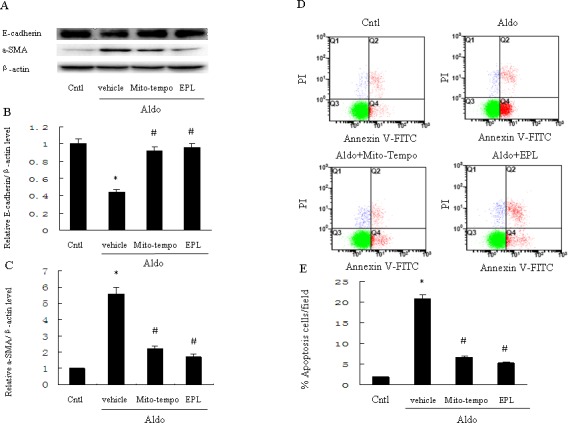
Treatment with Mito-Tempo or EPL inhibits Aldo-induced cell phenotypic alternation and apoptosis in HK-2 cells HK-2 cells were pretreated with Mito-Tempo (10 μM) or EPL (10 μM) for 30 min, followed by incubation with Aldo (10^−7^ M) for 48 h. **A.** Western blot of E-cadherin and α-SMA. **B.**, **C.** Densitometric analysis of E-cadherin (B) and α-SMA(C). **D.**, **E.** HK-2 cell apoptosis measured by flow cytometry. Cells were pretreated with Mito-tempo (10 μM) and EPL (10 μM) for 30min, followed by incubation with Aldo (10^−7^ M) for 24 h. Data are expressed as mean ± SEM (*n* = 6). *, *P* < 0.01 *vs*. Cntl; ^#^, *P* < 0.01 *vs*. Aldo-treated group.

To determine the role of mitochondrial ROS in Aldo-induced epithelial tubular injury, we examined apoptosis and phenotypic alternation in HK-2 cells following stimulation with Aldo. Aldo stimulation significantly reduced renal E-cadherin and significantly increased renal α-SMA (Figure 8A-8C). However, treatment with Mito-Tempo significantly reversed these Aldo-induced patterns of protein expression (Figure 8A-8C). Similarly, tubular cell apoptosis was significantly increased in the Aldo-treated group compared to the control group, and the induction of apoptosis by Aldo was markedly inhibited by Mito-Tempo (Figure 8D, 8E). EPL also markedly inhibited Aldo-induced phenotypic alternation and apoptosis of HK-2 cells (Figure 8A-8E).

### Mitochondrial ROS triggers NLRP3 inflammasome activation in Aldo-induced HK-2 cell injury

**Figure 9:**
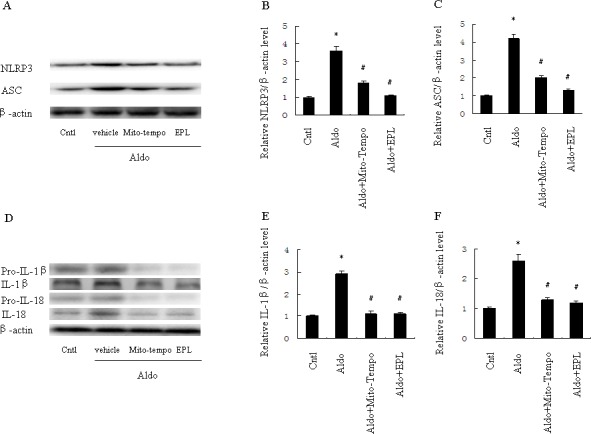
Treatment with Mito-Tempo or EPL inhibited Aldo-induced activation of the NLRP3 inflammasome in HK-2 cells Cells were pretreated with Mito-tempo (10 μM) or EPL (10 μM) for 30 min, followed by incubation with Aldo (10^−7^ M) for 48 h. **A.** Western blot of NLRP3 and ASC. **B.**, **C.** Densitometric analysis of NLRP3 (B) and ASC (C). **D.** Western blot of IL-1β and IL-18. **E.**, **F.** Densitometric analysis of IL-1β (E) and IL-18 (F). Data are expressed as mean ± SEM (*n* = 6). *, *P* < 0.01 *vs*. Cntl; ^#^, *P* < 0.01 *vs*. Aldo-treated group.

To determine whether Aldo-induced NLRP3 inflammasome activation was mediated by mitochondrial ROS in HK-2 cells, we evaluated the NLRP3 inflammasome and its downstream effectors, ASC, IL-1β and IL-18. The expression of NLRP3, ASC, and mature IL-1β and IL-18 protein were significantly increased in HK-2 cells following Aldo stimulation; treatment with Mito-Tempo markedly inhibited these protein expression patterns. Similarly, EPL also prevented NLRP3 inflammasome activation in HK-2 cells in response to Aldo stimulation (Figure 9A-9F).

## DISCUSSION

Although a large number of researches suggest that Aldo plays a pathogenic role in the development of kidney disease, the underlying mechanism of Aldo-induced CKD remains incompletely understood. In present study, we demonstrate that Aldo induces renal tubular injury through mitochondrial ROS-mediated NLRP3 inflammasome activation. Our data suggest that the NLRP3 inflammasome may serve as a therapeutic target in the prevention or treatment of Aldo-induced tubular injury.

Inflammation is part of the normal response to tissue damage. Recently, the NLR family of pattern recognition receptors has been shown to mediate various disease processes *via* the regulation of pro-inflammatory cytokines [18]. Komada *et al.* demonstrated that inflammasome related molecules were increased in the kidney of a UUO model, and that extracellular adenosine triphosphate triggered inflammasome activation in epithelial cells of the renal collecting duct through ROS-dependent signals [19]. Liu *et al.* also demonstrated that the NLRP3 and IL-18 proteins are localized in the proximal tubular cells in kidney biopsy specimens from patients with IgA nephropathy; these NLRP3 inflammasome levels were closely correlated with the extent of proteinuria and tubulointerstitial fibrosis [20, 21]. In the present study, we found that Aldo increased the activation of the NLRP3 inflammasome and its downstream cytokines in a dose- and time-dependent manner. Moreover, silencing of the NLRP3 inflammasome significantly reversed Aldo-induced apoptosis and phenotypic alternation *in vitro*, suggesting that the NLRP3 inflammasome plays a key role in the progression of renal tubular cells injury.

NLRP3 is a detrimental factor in some models of proteinuric or nonproteinuric kidney injury. In these studies, NLRP3 knockout (KO, NLRP3^−/−^) mice were resistant to kidney injury *via* inhibiting inflammatory processes [22-24]. In the present study, we characterized the role of the NLRP3 inflammasome in mediating inflammatory responses in Aldo-induced renal tubular injury. We found that indicators of kidney damage, including apoptosis and phenotypic alternation of renal tubular cells, were attenuated in Aldo-infused NLRP3^−/−^ mice compared with Aldo-infused WT mice. Furthermore, the NLRP3 inflammasome and downstream cytokines IL-1β and IL-18 were also inhibited in Aldo-infused NLRP3^−/−^ mice. Taken together, these data suggest that the NLRP3 inflammasome plays a key role in renal injury triggered by Aldo *in vivo*.

IL-1β and IL-18 are two inflammatory cytokines implicated in various kinds of CKD, and several studies demonstrated that IL-1β and IL-18 could induce expression of mesenchymal markers in tubular epithelial cells [25, 26]. It has also been demonstrated that IL-1β and IL-18 are significantly increased in Aldo-induced renal inflammation and fibrosis *in vivo* [27, 28], suggesting that IL-1β and IL-18 may be the key mediators of Aldo-induced renal injury, although the exact mechanisms remained unclear. In present study, we demonstrate that Aldo stimulation induces the production of mature IL-1β/IL-18 in renal tubular cells in a dose- and time-dependent manner. Moreover, we show that silencing of the NLRP3 inflammasome in HK-2 cells significantly decreased expression of mature IL-1β and IL-18, and reduced apoptosis. Similarly, mature IL-1β and IL-18 were markedly decreased in kidney samples from Aldo-infused NLRP3^−/−^ mice, indicating that the absence of NLRP3 ameliorates renal tubular injury and improves renal function, and suggesting that NLRP3 inflammasome-dependent mature IL-1β/IL-18 release may be implicated in renal tubular inflammation and injury. These data suggest an important role for the NLRP3 inflammasome/IL-1β/IL-18 axis in the progression of Aldo-induced renal injury.

ROS function as key signaling molecules and have been shown to be essential for proliferation and migration of renal tubular cells. Interestingly, ROS was also shown to mediate the downstream mechanism of Aldo-induced renal tubular cells injury [2, 17]. Swerdlow *et al.* demonstrated that excessive ROS production was closely linked to mitochondrial function [29]. Furthermore, accumulation of mitochondrial ROS was sufficient to activate the NLRP3 inflammasome [14, 30, 31]. However, the role of the mitochondrial ROS/NLRP3 inflammasome axis was still unclear in Aldo-induced renal injury. Mito-Tempo is a nitroxide linked to a positively charged triphenyl phosphonium cation, and is targeted specifically to the mitochondria [32]. Mito-Tempo treatment markedly abolishes mitochondrial ROS production, caspase-1 activation, and mtDNA translocation in response to LPS and ATP [33]. Notably, in our present study we also found that the production of mitochondrial ROS was significantly increased in response to Aldo stimulation of HK-2 cells *in vitro*. Treatment with Mito-Tempo markedly abolished mitochondrial ROS, decreased renal tubular cell apoptosis, and attenuated phenotypic alteration associated with Aldo-stimulation of HK-2 cells. Furthermore, Mito-Tempo significantly inhibited the NLRP3 inflammasome and its relative downstream cytokines. These findings support the growing importance of the mitochondrial ROS-activated NLRP3 inflammasome in Aldo-induced renal tubular cell injury.

MR was suggested to mediate the pathogenic role of Aldo in a model of CKD. Recent reports suggest the anti-fibrotic and anti-inflammatory effect of an MR antagonist in the progression of CKD [34, 35]. In present study, we demonstrate that Aldo induces mitochondrial oxidative stress and apoptosis in renal tubular cells *via* an MR-dependent mechanism. Furthermore, Aldo stimulation induced the activation of the NLRP3 inflammasome and its downstream cytokines, which was dependent on excessive mitochondrial ROS. Blockade of MR with EPL significantly prevented mitochondrial ROS production and activation of the NLRP3 inflammasome. Taken together, these results indicate that MR is responsible for the pro-inflammatory effect of Aldo in renal tubular cells.

In summary, our study demonstrates that Aldo induces NLRP3 inflammasome activation *via* MR *in vitro* and *in vivo*. Mitochondrial ROS are involved in Aldo-induced renal tubular injury, and may act as redox-signaling messengers. The mitochondrial-targeted antioxidant significantly inhibited oxidative stress, activation of the NLRP3 inflammasome, and attenuated HK-2 cell injury following treatment with Aldo. These results support the concept that mitochondrial ROS triggers the activation of the NLRP3 inflammasome, leading to Aldo-induced renal injury. Our characterization of the mitochondrial/NLRP3 inflammasome axis may provide new insight into the pathogenic progression of Aldo-induced kidney damage.

## MATERIALS AND METHODS

### Reagents and antibodies

Aldo, Eplerenone (EPL), Mito-Tempo, and 2′,7′-dichlorofluorescein diacetate were purchased from Sigma (St. Louis, MO). MitoSOX^TM^ Red reagent, an indicator of mitochondrial superoxide, was purchased from Invitrogen (Carlsbad, CA). Rabbit polyclonal anti-E-cadherin, goat polyclonal anti-cleaved caspase-1 p20 antibody, Rabbit polyclonal anti-IL-18, and mouse monoclonal anti-caspase-1 antibody were purchased from Santa Cruz Biotechnology (Santa Cruz, CA). Mouse monoclonal anti-NLRP3 antibody and rabbit polyclonal anti-ASC antibody were purchased from adipoGen company (San Diego, CA). Mouse monoclonal anti-α-SMA antibody was purchased from Abcam (Cambridge, MA). Goat polyclonal anti-IL-1β antibody was purchased from R&D Systems (Minneapolis, MN)

### Cell culture and transient transfection of HK-2 with NLRP3 small interfering RNA

HK-2 cells were maintained in DMEM/F12 medium, supplemented with 10% fetal bovine serum at 37°C and 5% CO2 in a humidified incubator. Transient transfection of HK-2 cells with siRNA was previously demonstrated [17]. HK-2 cells were grown to 60% confluence and then transfected with 500 nM NLRP3 siRNA or scramble siRNA (Santa Cruz, CA) using lipofectamine (Invitrogen, CA), prior to Aldo treatment.

### Western blot

HK-2 cells or renal samples were lysed and sonicated in cold PBS. 30μg of protein from the whole cell preparation were denatured in boiling water for 15 min, separated on SDS-PAGE gel, and transferred onto nitrocellulose membranes. Immunoblotting was performed with primary antibodies against NLRP3 (1:1000), ASC (1:1000), caspase-1 (1:500), cleaved caspase-1 (1:500), IL-1β (1:1000), IL-18 (1:1000), E-cadherin (1:1000), or α-SMA (1:500), followed by the addition of the appropriate HRP-labeled secondary antibodies. Membranes were probed with the enhanced chemiluminescent system (KPL, Gaithersburg, MD) for visualization of protein bands, and densitometric analysis was performed using previously described methods [17].

### Apoptosis detection with Annexin V/propidium iodide staining and Hoechst 33258 staining

Annexin V is a Ca^2+^-dependent phospholipid binding protein with a high affinity for phosphatidylserine, which is externalized on the surface of the cell membrane during the progression of apoptosis. Detection of apoptosis in HK-2 cells according to the Annexin V/propidium iodide method (BD Biosciences, San Diego, CA) was performed as previously described [17]. After treatment, HK-2 cells were seeded into six-well plates and indicators of apoptosis were determined on a FACScan flow cytometer (Epics Altra, Beckman Coulter, CA) according to the manufacturer's instructions. For detecting apoptosis with Hoechst 33258, HK-2 cells were grown on glass coverslips and were staining with Hoechst 33258 after treatment; staining was then analyzed by fluorescence microscopy.

### Animal studies

All animal experiments were performed according to the approval of the animal care committee at the Jiaotong University. C57BL/6J (wild type, WT) mice were obtained from Shanghai SLAC Laboratory Animals (Shanghai, China). NLRP3^−/−^ mice (C57BL/6J genetic background) were purchased from the Jackson Laboratory (Sacramento, CA). All mice underwent left uninephrectomy under anesthesia with sodium pentobarbital. After 10 days of recovery from surgery, mice were given high-salt drinking water (containing 1% sodium chloride). An osmotic mini-pump (Alzet model 2004) was implanted subcutaneously to infuse Aldo (0.75 μg/h) or vehicle control (0.5% ethanol) for 4 weeks. Mice were allowed to recover with free access to food and drinking water. All mice were sacrificed at week 4, whereupon plasma samples were collected. At sacrifice, sections of kidney were harvested and fixed in 10% formalin, followed by embedding in paraffin for histological evaluation. The remainder of the kidney was used for mRNA and protein analysis. Serum creatinine, blood urea nitrogen (BUN), and excreted levels of urinary albumin and creatinine were measured by automatic analyzers (Hitachi, Tokyo, Japan). Serum IL-1β and IL-18 were measured with ELISA kits (RayBiotech, Norcross, GA) according to the manufacturer's instruction.

### Terminal deoxyribonucleotide transferase (TdT)-mediated dUTP nick-end labeling (TUNEL) assay

Apoptosis in cultured cells or kidney tissue sections were identified using the TUNEL assay. In brief, deparaffinized kidney sections were treated in a permeabilization solution (0.1% Triton X-100 in 0.1% sodium citrate) at 4°C for 2 min. After washing twice with PBS, the deparaffinized sections were incubated with 50 μl TUNEL reaction mixture for 60 min at 37°C in the dark. For quantification, 10-20 fields were randomly selected from each tissue section, and the number of TUNEL-positive cells was counted per millimeter.

### ROS and mitochondrial superoxide measurement

The fluorogenic substrate DCFDA was used to detect the whole intracellular production of ROS, as previously described. To measure ROS levels, HK-2 cells were seeded into six well plates, and allowed to grow until they reached confluent cell density. Plates were treated with 10 μl DCFDA for 30 min at 37°C in the dark. HK-2 cells were then washed twice, and fluorescence was measured using a fluorescence plate reader at the excitation and emission wavelengths of 485 and 528 nm, respectively [17]. Mitochondrial ROS were also detected with MitoSOX^TM^ Red reagent. Briefly, six well plates were treated with MitoSOX^TM^ Red (5μM) for 30 min at 37°C in the dark. For the inhibition of mitochondrial ROS, Mito-Tempo (10μM) or EPL (10μM) were added for an additional 15 min. Fluorescence was then measured as described above.

### Statistical analysis

Data are presented as mean ± the standard error of the mean (SEM). One-way ANOVA was used to compare mean values; results with *P* < 0.05 were considered significant.
